# Unraveling the neuroprotective mechanisms of elephant black garlic extract against beta amyloid peptide-induced neurotoxicity

**DOI:** 10.3389/fnut.2025.1725284

**Published:** 2026-01-16

**Authors:** Javiera Gavilan, Jessica Panes-Fernández, Aníbal Araya, Claudia Pérez-Manríquez, José Becerra, Patricio Varas, Gustavo Moraga-Cid, Gonzalo E. Yévenes, Jorge Fuentealba

**Affiliations:** 1 Departamento de Fisiología, Facultad de Ciencias Biológicas, Universidad de Concepción, Concepción, Chile; 2 Laboratorio de Química de Productos Naturales, Facultad de Ciencias Naturales y Oceanográficas, Universidad de Concepción, Concepción, Chile; 3 Agrícola Melimei, Ancud, Chile; 4 Millennium Nucleus for the Study of Pain (MiNuSPain), Concepción, Chile; 5 Centro de Investigaciones Avanzadas en Biomedicina (CIAB-UdeC), Universidad de Concepción, Concepción, Chile

**Keywords:** aged garlic, *Allium ampeloprasum*, Alzheimer’s disease, mitochondrial dysfunction, neuroprotection, soluble oligomers of beta-amyloid peptide (SO-aβ), sulphurated compounds, synaptic failure

## Abstract

Chilean Elephant Black Garlic (*Allium ampeloprasum*) is a distinctive variety whose biological activity remains largely unexplored. Unlike conventional black garlic derived from *Allium sativum*, this preparation exhibits a markedly different chemical profile enriched in sulfur-containing metabolites such as 3H-1,2-dithiol-3-thione (D3T) and 4-methyl-1,2,3-trithiolane (TTL). These compounds are rarely detected in traditional black garlic extracts and may underlie the specific protective effects observed in this study. Consequently, the biological actions described here cannot be generalized to other black garlic preparations. We provide the first evidence that Chilean Elephant Black Garlic (BG) confers neuroprotection against the toxicity of soluble oligomers of amyloid-beta (SO-Aβ), a central pathogenic element of Alzheimer’s disease (AD) known to trigger synaptic dysfunction and mitochondrial impairment. In mouse hippocampal slices, BG prevented SO-Aβ-induced reductions in cell viability. Complementary experiments in cultured hippocampal neurons showed that BG preserved spontaneous calcium signaling and prevented SO-Aβ-mediated alterations in neuronal arborization. At the molecular level, BG restored the expression of synaptic proteins (SV2 and PSD-95) and maintained BDNF levels. Beyond synaptic protection, BG also preserved mitochondrial function under SO-Aβ challenge by maintaining mitochondrial membrane potential, intracellular ATP levels, and Mitofusin-1 expression. Altogether, these findings identify Chilean Elephant Black Garlic, as functional food, that represent an important source of bioactive compounds with properties capable of counteracting key neurotoxic mechanisms implicated in AD.

## 1 Introduction

Alzheimer’s disease (AD) is characterized by a progressive neuronal decline in the hippocampus and cortex (1). The amyloid-beta (Aβ) hypothesis proposes that soluble Aβ oligomers (SO-Aβ) are major drivers of synaptic dysfunction and neuronal injury (2, 3). SO-Aβ have been shown to impair synaptic function by reducing vesicular presynaptic proteins such as dynamin-1, synapsin-1, and SV2 (4, 5), as well as postsynaptic proteins including PSD-95 and NMDA receptors (6). In addition, SO-Aβ disrupt mitochondrial homeostasis by decreasing mitochondrial membrane potential (ΔΨm), oxidative phosphorylation, and ATP production (7), and by altering mitochondrial fusion/fission dynamics (8). Collectively, these events diminish neuronal network activity and lead to chronic synaptic failure (9). Current AD treatments, such as donepezil and memantine, provide only symptomatic relief and do not halt disease progression (10). Recently, the FDA approved monoclonal antibodies targeting Aβ aggregates (aducanumab, lecanemab), but their clinical efficacy remains under critical evaluation (11), underscoring the need for complementary therapeutic strategies.

Common garlic (*Allium sativum*) has been widely studied, demonstrating diverse health benefits across preparations (12). Aged garlic extract (AGE, also known as black garlic) is obtained by exposing fresh garlic to controlled humidity, temperature, and time (13, 14), which increases the abundance of bioactive molecules such as phenolic compounds and organosulfur metabolites (15). Experimental evidence shows that AGE prevents Aβ-induced cognitive decline, improving short-term recognition memory (16), as well as working and reference memory (17). In Tg2576 mice, AGE or S-allyl-cysteine (SAC), its main organosulfur constituent, reduces Aβ accumulation in cortex and hippocampus (18) and stabilizes presynaptic proteins affected by Aβ toxicity, including SNAP-25 and synaptophysin (19). Mechanistic studies suggest that AGE/SAC may modulate neuronal gene expression (20), improve endoplasmic reticulum stress (21), and inhibit caspase-3 activity (22).

Despite extensive characterization of *Allium sativum*–derived black garlic, little is known about the biological activity of Elephant Black Garlic obtained from *Allium ampeloprasum*, a variety native to Chiloé Island (Chile). This preparation displays a distinct chemical profile enriched in uncommon sulfur-containing metabolites such as 3H-1,2-dithiol-3-thione (D3T) and 4-methyl-1,2,3-trithiolane (TTL), which are largely absent from conventional AGE. We recently reported that *A. ampeloprasum* black garlic (BG) contains unique bioactive molecules with strong antioxidant and neuroactive properties (14).

Given its distinctive phytochemical composition, Chilean Elephant Black Garlic represents an unexplored functional food or nutraceutical candidate with potential neuroprotective properties that remain poorly understood. In this study, we investigated the effects of BG in ex vivo and *in vitro* models of SO-Aβ toxicity, with a particular focus on synaptic and mitochondrial function. This approach is essential for defining the translational potential of this unique AGE preparation and its bioactive constituents in the context of neurodegenerative diseases.

## 2 Materials and methods

### 2.1 Preparation of black garlic extract

Preparation of BG from fresh elephant garlic cloves was performed by Melimei Agricultural Company (Manao, Chiloé Island, Chile). Briefly, fresh garlic cloves of *Allium ampeloprasum* were subjected to an aging process. A temperature of 70 °C and 90% humidity were applied for a period of 20 consecutive days (13, 14, 23). During this process, cloves were arranged in a single layer within temperature–humidity-controlled chambers, and environmental parameters were continuously monitored to maintain deviations below ±1 °C and ±2% relative humidity. No additives or fermentation starters were used, ensuring that aging depended exclusively on controlled thermal–humidity conditions.

To prepare the garlic extract, 50 g of chopped BG cloves were subjected to maceration in methanol (MeOH; HPLC grade) under magnetic stirring for 48 h at room temperature (RT) (ca. 19 °C).

Subsequently, the extract was centrifuged at 4,000 g for 20 min, and the supernatant was filtered through polycarbonate 0.45-μm pore-size membranes (Merck Chemicals). The extract was concentrated in a rotary evaporator (Heidolph WB 2001), avoiding light exposure, then lyophilized and stored in the dark at 4 °C. For extract standardization, each batch was analyzed by GC–MS to verify the presence of characteristic organosulfur markers previously identified for this preparation (e.g., 5-HMF, DAS, DADS, D3T, and TTL), ensuring qualitative consistency across experiments (14). Concentrations used in biological assays were normalized according to dry-weight yield (mg extract/mL). For biological tests, the extract was suspended in phosphate-buffered saline (PBS 1X, pH 7.4, Corning) to the desired concentration.

### 2.2 Soluble oligomers of aβ (SO-aβ) preparation

The lyophilized Aβ_1–40_ peptide (GenicBio, Shanghai, China) stock was reconstituted in dimethyl sulfoxide (DMSO) at 2.3 mM and stored at −20 °C. Two-microliter aliquots were dissolved in PBS 1X to obtain an 80 μM solution. To generate oligomeric forms, the solution was subjected to vertical stirring (500 rpm) using a magnetic agitator for 4 h at room temperature. These aggregates were previously characterized (8). SO-Aβ was used at 0.5 μM in PC-12 cells and primary hippocampal neuron cultures, and at 2.5 μM in hippocampal slices.

### 2.3 Cell culture

Cell culture procedures were previously described (24). Briefly, PC-12 cells derived from adrenal gland pheochromocytoma (CRL-1721.1, ATCC, Manassas, VA, United States) were cultured in Dulbecco’s Modified Eagle Medium (DMEM) supplemented with 5% fetal bovine serum (FBS), 5% horse serum (HS), penicillin (100 U/mL), streptomycin (100 μg/mL), and L-glutamine (2 mM). Cells were plated at 100,000 cells/well, incubated under standard conditions (37 °C, 5% CO₂), and used after 24 h.

Primary cultures of embryonic hippocampi (E18) were plated at 320,000 cells/mL on coverslips coated with poly-L-lysine (Trevigen, Gaithersburg, MD, United States) in Minimal Essential Medium (MEM; Gibco) supplemented with HS (10%), DNase (4 mg/mL), and L-glutamine (2 mM) for 24 h. The culture medium was replaced after 24 h with MEM containing HS (2%), FBS (2%), and N3 supplement (0.5%; BSA 1 mg/mL, putrescine 4 mg/mL, insulin 1.25 mg/mL, sodium selenite 1 μg/mL, T₃ 2 μg/mL, progesterone 1.25 μg/mL, corticosterone 4 μg/mL). Cell cultures were maintained at 37 °C with 5% CO₂, and experiments were performed after 10–11 days *in vitro* (DIV).

C57BL/6 mice were treated in accordance with NIH regulations and the Bioethics Committee of the University of Concepción (permission CEBB 667-2020). The study adhered to the ARRIVE guidelines for reporting animal research. Mice were deeply anesthetized using CO₂ inhalation before euthanasia by cervical dislocation.

### 2.4 Preparation of hippocampal slices

Mice (C57BL/6, 3–4 months old, 20–25 g) were anesthetized with isoflurane. Brains were quickly removed from the skull and placed in a cutting solution composed of NaCl 120 mM, KCl 1 mM, CaCl₂ 0.5 mM, NaHCO₃ 26 mM, MgSO₄ 10 mM, KH₂PO₄ 1.18 mM, glucose 11 mM, and sucrose 200 mM (pH 7.4). Brains were mounted on a Microslicer (DTK-1000, Ted Pella, Redding, CA, United States) to obtain 200-μm-thick slices. Subsequently, hippocampi were isolated and transferred to artificial cerebrospinal fluid (aCSF: NaCl 120 mM, KCl 2 mM, CaCl₂ 2 mM, NaHCO₃ 26 mM, MgSO₄ 1.19 mM, KH₂PO₄ 1.18 mM, glucose 11 mM; pH 7.4) in a water bath (34 °C, 1 h). Finally, slices were superfused with aCSF and treated with black garlic extract (BG; 20 μg/mL), SO-Aβ (2.5 μM), SO-Aβ + BG, or the mitochondrial uncoupler FCCP (10 μM) for 3 h. In all experiments, solutions were continuously bubbled with a 95% O₂/5% CO₂ gas mixture. All procedures involving animals were conducted in accordance with ARRIVE guidelines and approved institutional protocols.

### 2.5 Cell viability assay

To evaluate changes in cell viability, we used the MTT assay based on 3-(4,5-dimethylthiazol-2-yl)-2,5-diphenyltetrazolium bromide (MTT, Sigma-Aldrich) (25). This method measures the ability of mitochondria to reduce MTT to insoluble formazan. PC-12 cells subjected to the different experimental conditions were incubated for 30 min with MTT (1 mg/mL). Hippocampal slices were incubated with MTT (0.5 mg/mL) for 1 h at 37 °C. PBS 1X (Gibco) was used to dilute MTT. Formazan crystals were solubilized with 100 μL of 2-propanol, and absorbance at 560 and 620 nm was measured in a 96-well plate using a NOVOstar multiplate reader (BMG Labtech, Ortenberg, Germany).

### 2.6 Spontaneous Ca^2+^ signals

Hippocampal cultures (10–11 DIV) were incubated with the Ca^2+^-sensitive fluorescent probe Fluo-4 a.m. (5 μM; Invitrogen, Carlsbad, CA, United States) for 20 min in PBS 1X (26). Neurons were then washed with PBS 1X for 20 min, followed by two washes with standard external solution (SEN), and subsequently imaged under an inverted microscope. Fluorescence changes (excitation: 480 nm; emission: 520 nm) were acquired with an exposure time of 200 ms, at 1-s intervals for 10 min, using an iXon+ EMCCD camera (Andor, Belfast, Ireland) and Imaging Workbench 6.0 software (Indec Biosystems, Burlington, ON, Canada). Intracellular Ca^2+^ signals were measured after 24 h of treatment.

### 2.7 Immunofluorescence

Cell cultures were fixed with 4% paraformaldehyde for 15 min at 4 °C, followed by permeabilization and blocking with 10% HS and 0.1% Triton X-100 for 20 min at RT. Primary antibodies, including SV2 (mouse, Hybridoma Bank), PSD95 (mouse, Antibodies Incorporated), BDNF (rabbit, Santa Cruz Biotechnology), MFN1 and DRP1 (rabbit, Novus Biologicals), MAP2 (guinea pig, Synaptic Systems), and MAP1B (goat, Santa Cruz Biotechnology), were applied at 1:200 and incubated for 1 h at RT. The corresponding secondary antibodies—anti-mouse and anti-rabbit (Alexa Fluor 488, Jackson ImmunoResearch), anti-rabbit and anti-goat (Cy3, Jackson ImmunoResearch), and anti-guinea pig (Alexa Fluor 647, Progen)—were used at 1:200 and incubated for 1 h at RT. Preparations were mounted using fluorescence mounting medium (Dako, Glostrup, Denmark), and images were acquired using spectral confocal microscopy (LSM780 NLO, Zeiss) or epifluorescence microscopy (Nikon Eclipse Ts2). Image processing was performed with ImageJ 1.54f (NIH, Bethesda, Maryland, United States). For quantification, four regions of interest (15 × 15 px) were selected in each image, using MAP2 or MAP1B as neuronal markers to ensure unbiased selection; fluorescence intensity was plotted for these regions.

### 2.8 Western blot

Slices were mechanically lysed in a homogenization buffer containing 10 mM Tris–HCl (pH 7.4), 10 mM N-ethylmaleimide, 0.25 M sucrose, and protease inhibitors, and centrifuged at 8,000 g for 10 min at 4 °C to obtain the supernatant. Electrophoresis was performed on 10% polyacrylamide gels (100 V, 100 min), and proteins were transferred to PVDF membranes (250 mA, 120 min). Membranes were blocked with 5% milk for 1 h, and primary antibodies were incubated overnight at 4 °C: SV2 (1:1,000, mouse, Hybridoma Bank), PSD95 (1:1,000, mouse, Antibodies Incorporated), MFN1 and DRP1 (1:1,000, rabbit, Novus Biologicals), and *β*-actin (1:1,000, mouse, Santa Cruz Biotechnology). Secondary antibodies—anti-mouse-HRP and anti-rabbit-HRP (1:5,000, Santa Cruz Biotechnology)—were incubated for 1 h at RT. Immunoreactive bands were detected using the Clarity™ Western ECL substrate (Bio-Rad, United States) and visualized with the Odyssey imaging system (LI-COR, Lincoln, NE, United States). Quantification of band intensity was performed with Image Studio software (Image Studio Inc., Appleton, WI, United States).

### 2.9 Automated Sholl analysis

Neuronal morphology was analyzed using Bonfire v1.0 (Firestein Lab, Newark, New Jersey, United States), a semi-automated platform for dendritic and axonal analysis that integrates open-source tools. Neurons were traced in ImageJ (NeuronJ plugin), and connectivity between neurites was defined using NeuronStudio 0.9.92 (Susan L. Wearne, New York, United States). Bonfire employs custom MATLAB 2015 (MathWorks, Natick, Massachusetts, United States) scripts to convert data into the appropriate format, perform error checking, and carry out Sholl analysis (27).

### 2.10 Measurement of mitochondrial membrane potential (ΔΨm)

Hippocampal slices were incubated with the JC-1 probe (Invitrogen; 2 μM) in aCSF for 30 min at 37 °C. After three washes with aCSF, slices were treated with SO-Aβ (2.5 μM), BG (20 μg/mL), SO-Aβ + BG, or FCCP (10 μM). JC-1 accumulates in mitochondria in a potential-dependent manner, emitting red fluorescence (590 nm; excitation 520 nm) in its aggregated form. In the cytoplasm, monomeric JC-1 emits green fluorescence (520 nm; excitation 485 nm). Thus, mitochondrial depolarization is indicated by a decrease in the red/green fluorescence ratio (28). Both wavelengths were acquired simultaneously for 3 h at 37 °C in a NOVOstar multiplate reader (black 96-well plate). The 590/520 ratio was used to quantify ΔΨm variations for each treatment.

### 2.11 ATP quantification

Hippocampal slices were treated for 3 h with SO-Aβ (2.5 μM), BG (20 μg/mL), SO-Aβ + BG, gramicidin (GRA; 100 μg/mL), or FCCP (10 μM). To measure intracellular ATP, slices were mechanically lysed in aCSF. The supernatant was used to quantify extracellular ATP. Ten microliters of each sample (lysates and supernatants) were loaded into a black 96-well microplate. To initiate the reaction, 90 μL of the ATP Determination Kit reagent (Invitrogen) was added. The reaction medium contained D-luciferin, recombinant firefly luciferase, and Mg^2+^, where luciferase converts luciferin to oxyluciferin in an ATP-dependent manner. Luminescence was detected at 560 nm using a NOVOstar multiplate reader (Labtech) at 28 °C (29). Background luminescence was subtracted from all measurements, and luminescence values were considered directly proportional to ATP concentration.

### 2.12 Statistical analysis

Experiments were performed with ≥3 independent biological replicates. Data are presented as mean ± SEM and expressed as percentage of the control. Statistical analysis was performed using an unpaired Student’s *t*-test for comparisons involving two groups, or one-way ANOVA followed by Dunnett’s *post hoc* test when comparing multiple treatments against a single control condition. Results *p < 0.05, **p < 0.01, and ***p < 0.001 versus control; #*p* < 0.05, ##*p* < 0.01, and ###*p* < 0.001 versus SO-Aβ were considered statistically significant. All analyses were conducted using GraphPad Prism 6.0 (Dr. Harvey Motulsky, Boston, Massachusetts, United States).

## 3 Results

### 3.1 Neuroprotective effects of BG on cellular models of chronic SO-aβ toxicity

**Figure 1:**
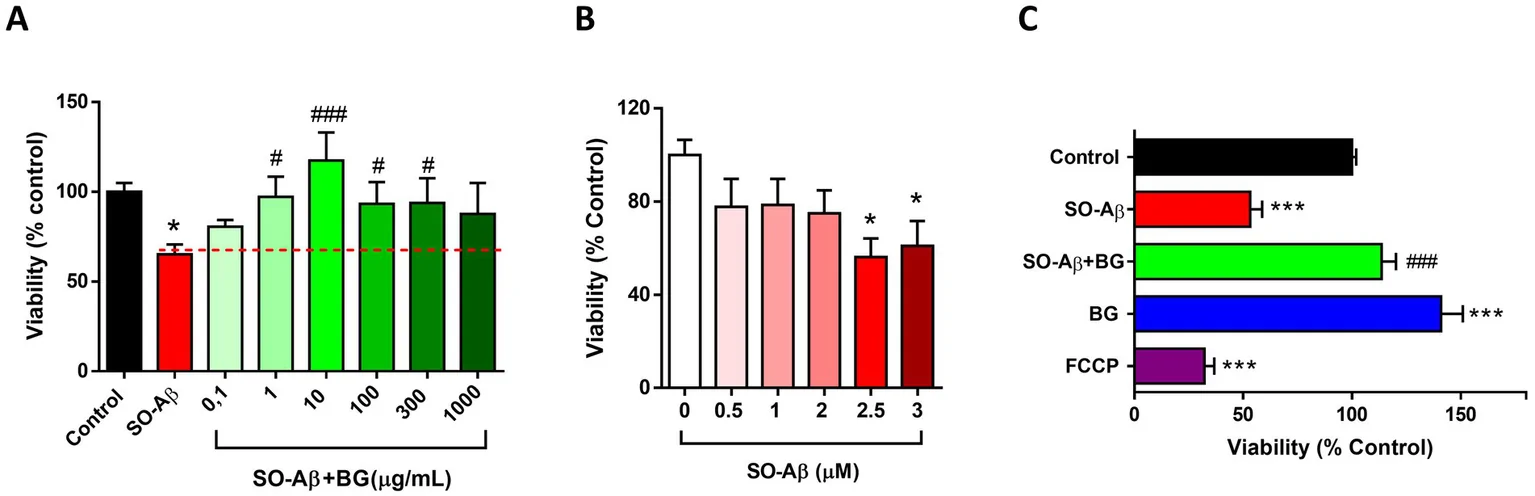
Neuroprotective effects of BG on cell viability under SO-Aβ toxicity. **(A)** PC-12 cells were treated with SO-Aβ (0.5 μM) and increasing concentrations of BG (0.1–1,000 μg/mL) for 24 h. Cell viability was measured using MTT method (*n* = 3, *N* = 9). **(B)** Hippocampal slices (200 μm thickness) from C57BL/6 mice were incubated for 3 h with increasing concentrations of SO-Aβ (0.5, 1, 2, 2.5, and 3 μM). Cell viability was measured using MTT method (*n* = 3, *N* = 9). **(C)** Slices were incubated for 3 h with SO-Aβ (2.5 μM) and BG (20 μg/mL) (*n* = 4; *N* = 12). FCCP (10 μM) was used as a positive toxicity control. Values are expressed as a percentage of the control without treatment *p < 0.05, ***p < 0.001 versus control; #*p* < 0.05 and ###*p* < 0.001 versus SO-Aβ.

To assess whether BG could prevent SO-Aβ toxicity, we first evaluated cell viability in PC-12 cells using the MTT assay. PC-12 cells were selected as the initial screening model because they provide a highly reproducible platform for quantifying Aβ-induced cytotoxicity and are widely used to establish dose–response relationships for natural extracts and organosulfur compounds, offering consistent sensitivity to oxidative and mitochondrial stress (8, 14, 24, 25, 30). Chronic exposure to SO-Aβ (0.5 μM, 24 h) reduced cell viability by 35 ± 5% compared to control conditions (100 ± 4%) (Figure 1A). Co-treatment with increasing concentrations of BG prevented SO-Aβ-induced toxicity in a dose-dependent manner, with values near control levels between 1 and 300 μg/mL [0.1: 80 ± 3%, 1: 97 ± 11%, 10: 117 ± 15%, 100: 93 ± 12%, 300: 93 ± 13%, 1,000: 87 ± 17% (μg/mL)] (Figure 1A). Based on this dose–response curve, we identified that 10 μg/mL produced the strongest and most consistent protective effect, and therefore this concentration was selected for all subsequent *in vitro* experiments (PC-12 and primary hippocampal neurons).

To validate BG’s protective effects in a system with greater structural and physiological preservation, we examined neuronal survival in acute hippocampal slices, which maintain neuronal–glial interactions and synaptic architecture. Using a range of SO-Aβ concentrations (0.5–3 μM, 3 h), we found that 2.5–3 μM SO-Aβ reduced slice viability by approximately 50%, establishing a suitable dynamic window to detect neuroprotection (Figure 1B). Considering that SO-Aβ (2.5 μM) produced a 47 ± 5% reduction in slice viability (Figure 1C), we selected 20 μg/mL BG because hippocampal slices are substantially thicker and contain a higher cellular density than monolayer cultures, requiring increased diffusion and penetration of the extract. Furthermore, slices were incubated for a shorter period (3 h vs. 24 h in cultured cells) and required a higher SO-Aβ concentration (2.5 μM vs. 0.5 μM) to reach comparable toxicity. Thus, doubling BG to 20 μg/mL ensured adequate tissue penetration and maintained proportionality with the increased toxic challenge. Under these conditions, BG fully restored slice viability to near-control levels (Control: 100 ± 2%; SO-Aβ + BG: 113 ± 6%). Interestingly, BG alone increased viability above control (40 ± 10%) (Figure 1C), consistent with a potential trophic action, as suggested previously (14). FCCP (10 μM) was used as a positive toxicity control and reduced slice viability by 68 ± 4%.

Altogether, these results justify the use of 10 μg/mL BG in cell-based systems and 20 μg/mL in hippocampal slices, reflecting differences in tissue architecture, incubation time, and SO-Aβ sensitivity across experimental models.

### 3.2 BG prevents SO-aβ-induced toxic effects on synaptic network

**Figure 2:**
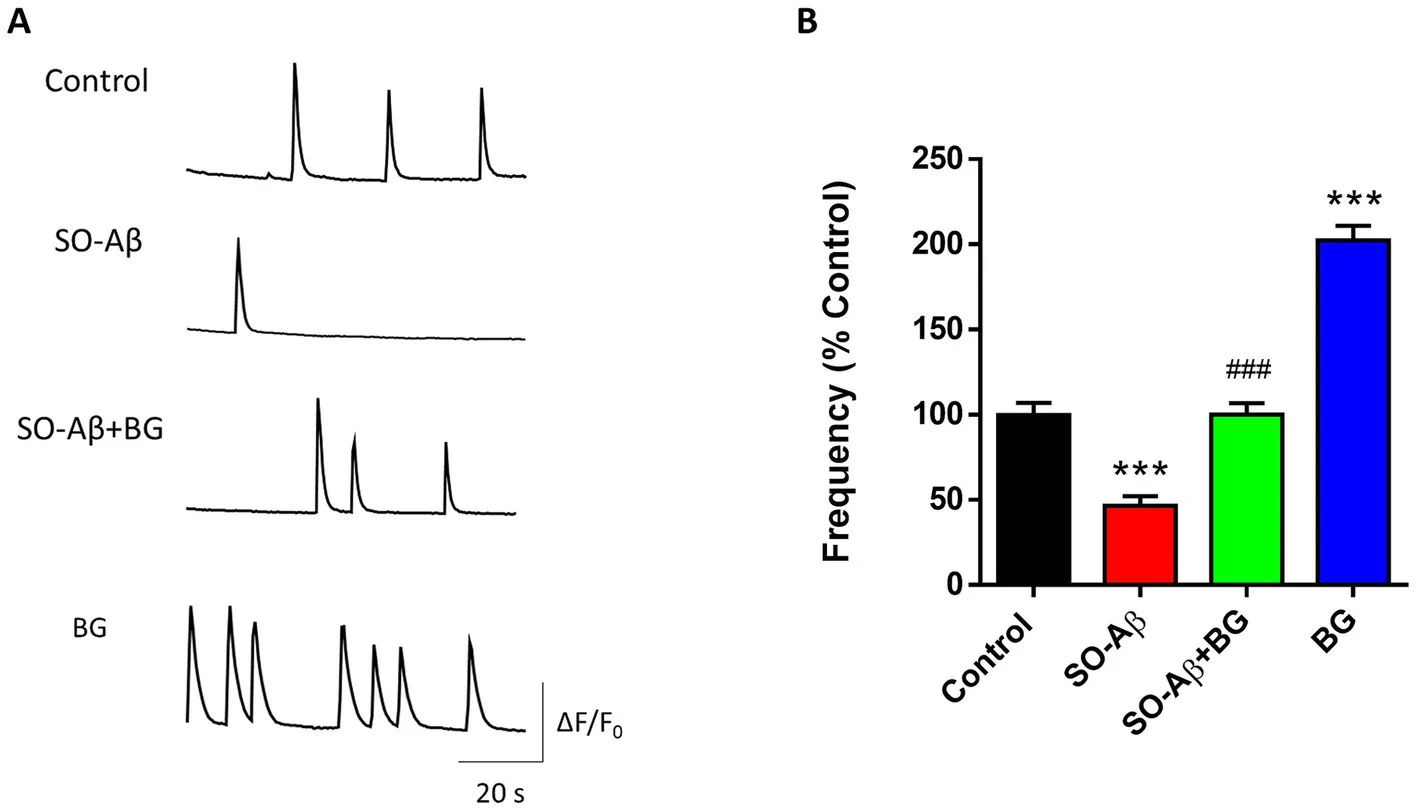
BG restores intracellular Ca^2+^ oscillations reduced by SO-Aβ. **(A)** Representative traces of spontaneous Ca^2+^ transients recorded from cultured hippocampal neurons. **(B)** Quantification of Ca^2+^ transient frequency after 24 h treatment with SO-Aβ (0.5 μM), BG (10 μg/mL) or SO-Aβ + BG (*n* = 3; *N* = 40). Values expressed as % of control, ***p < 0.001 versus control; ###*p* < 0.001 versus SO-Aβ.

In cultured hippocampal neurons, spontaneous intracellular Ca^2+^ transients reflect functional synaptic communication and exhibit characteristic frequency patterns. Our group has used this activity to assess neuronal network function and synaptic connectivity (25, 29, 31). Figure 2A shows representative recordings of these signals under the different experimental conditions. Chronic SO-Aβ treatment (0.5 μM, 24 h) reduced Ca^2+^ transient frequency to 54 ± 5% of control values (Figure 2B). We used 10 μg/mL BG in primary neurons because this was the optimal concentration identified in the PC-12 dose–response curve, and primary neurons—being more sensitive than cell lines—typically require lower concentrations to achieve maximal biological responses. Under these conditions, BG prevented the SO-Aβ-induced reduction in Ca^2+^ activity, restoring values comparable to controls (Control: 100 ± 7%; SO-Aβ + BG: 100 ± 6%). BG alone markedly increased Ca^2+^ transient frequency (202 ± 8%), indicating a strong enhancement of synaptic network activity.

**Figure 3:**
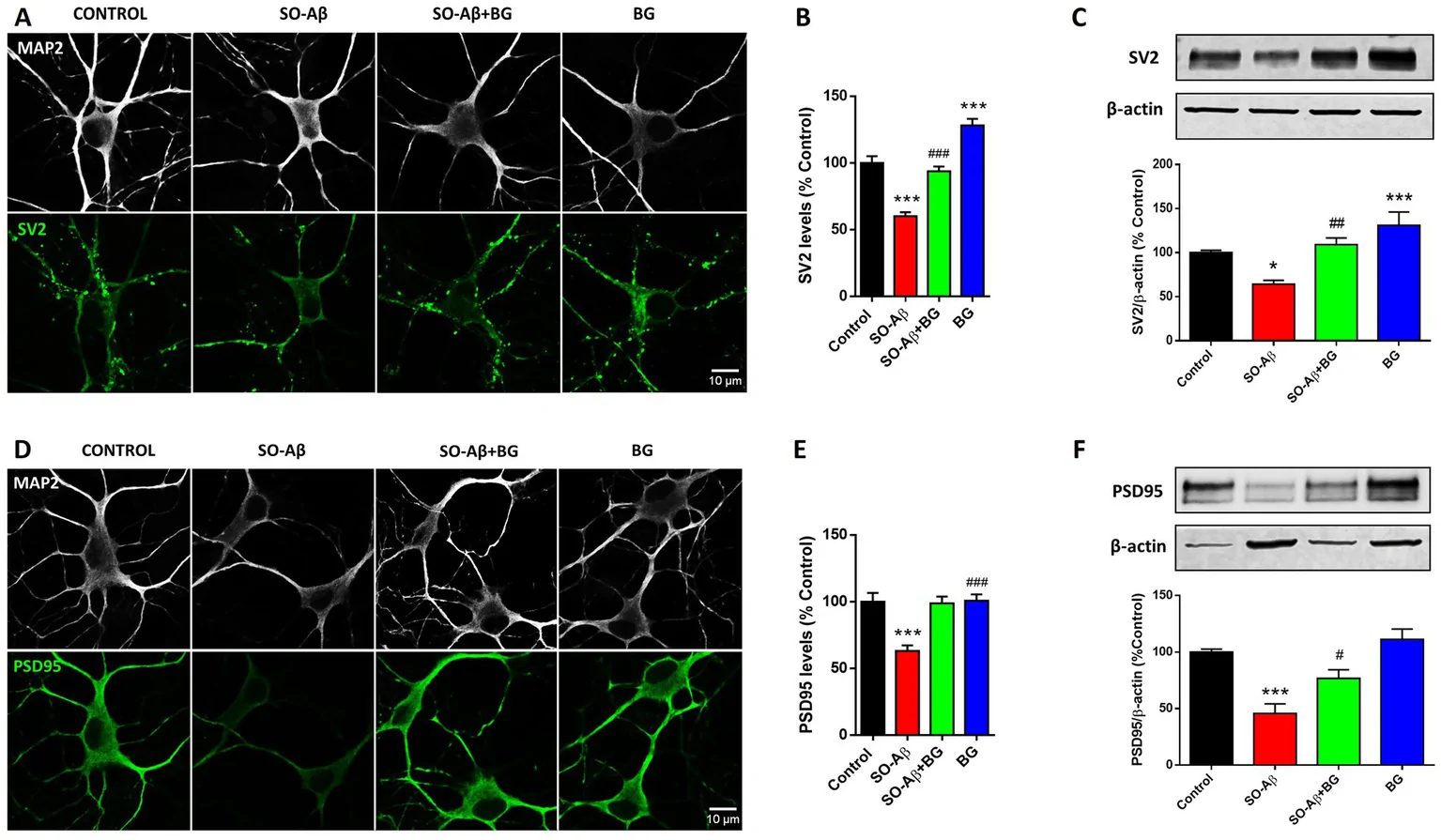
Effects of SO-Aβ and BG on synaptic protein levels of SV2 and PSD95. **(A)** Representative confocal images of hippocampal neurons treated for 24 h with SO-Aβ (0.5 μM), BG (10 μg/mL) and SO-Aβ + BG, and stained with specific antibodies for MAP2 (white), used as a neuronal marker, and for SV2 (green). Scale bar: 10 μm. **(B)** Quantification of SV2 immunofluorescence (*n* = 3; *N* = 15). **(C)** Western blot analysis of SV2 from hippocampal slices treated for 3 h with SO-Aβ (2.5 μM), BG (20 μg/mL) and SO-Aβ + BG. The quantification of the band intensities is plotted as a percentage of the control (*n* = 8). **(D)** Representative confocal images for PSD95 (green) and MAP2 (white) under the same conditions. Scale bar: 10 μm. **(E)** Quantification of PSD95 immunoreactivity (*n* = 3; *N* = 15). **(F)** Western blot quantification of PSD95 from slices (*n* = 8). The quantification of the band intensities is represented as a percentage of the control, *p < 0.05 and ***p < 0.001 versus control; #*p* < 0.05, ##*p* < 0.01, ###*p* < 0.001 versus SO-Aβ.

The beneficial effects of BG on cellular viability and Ca^2+^ signaling suggest that it reinforces synaptic structure and connectivity through both presynaptic and postsynaptic mechanisms. To explore this possibility, we evaluated the expression of the synaptic proteins SV2 and PSD95. SV2 is a key presynaptic vesicle protein (32) whose levels are reduced in AD patients and in cell models exposed to SO-Aβ (33). Figure 3A shows representative immunocytochemical images demonstrating the reduction in SV2 immunoreactivity caused by SO-Aβ (0.5 μM, 24 h) in hippocampal neurons. Quantification revealed that BG (10 μg/mL) preserved SV2 levels in the presence of SO-Aβ (Control: 100 ± 5%; SO-Aβ: 60 ± 2%; SO-Aβ + BG: 93 ± 3%), and additionally increased SV2 expression in control neurons (28 ± 5%) (Figure 3B). To further assess BG’s effects on synaptic proteins, we measured SV2 levels in hippocampal slices by western blot, using the same protocol of SO-Aβ treatments described in Figure 1. SO-Aβ treatment (2.5 μM, 3 h) reduced SV2 expression by 36 ± 4%, while BG (20 μg/mL) prevented this decrease (Control: 100 ± 2%; SO-Aβ + BG: 109 ± 7%) (Figure 3C). Consistent with the findings in cultured neurons, BG alone increased SV2 levels in slices (31 ± 15%).

We next evaluated PSD95, a postsynaptic scaffolding protein essential for maintaining synaptic plasticity (34, 35). SO-Aβ reduced PSD95 immunoreactivity in cultured hippocampal neurons (Figure 3D), as quantified in Figure 3E. As observed for SV2, BG (10 μg/mL) rescued PSD95 levels in the presence of SO-Aβ, restoring values comparable to controls (Control: 100 ± 6%; SO-Aβ: 63 ± 4%; SO-Aβ + BG: 98 ± 5%; BG: 100 ± 4%). Western blot analysis in hippocampal slices confirmed these effects (Figure 3F): BG restored PSD95 levels in SO-Aβ-treated slices (2.5 μM, 3 h) (C: 100%; SO-Aβ: 45 ± 8%; SO-Aβ + BG: 76 ± 7%, BG: 111 ± 9%).

**Figure 4:**
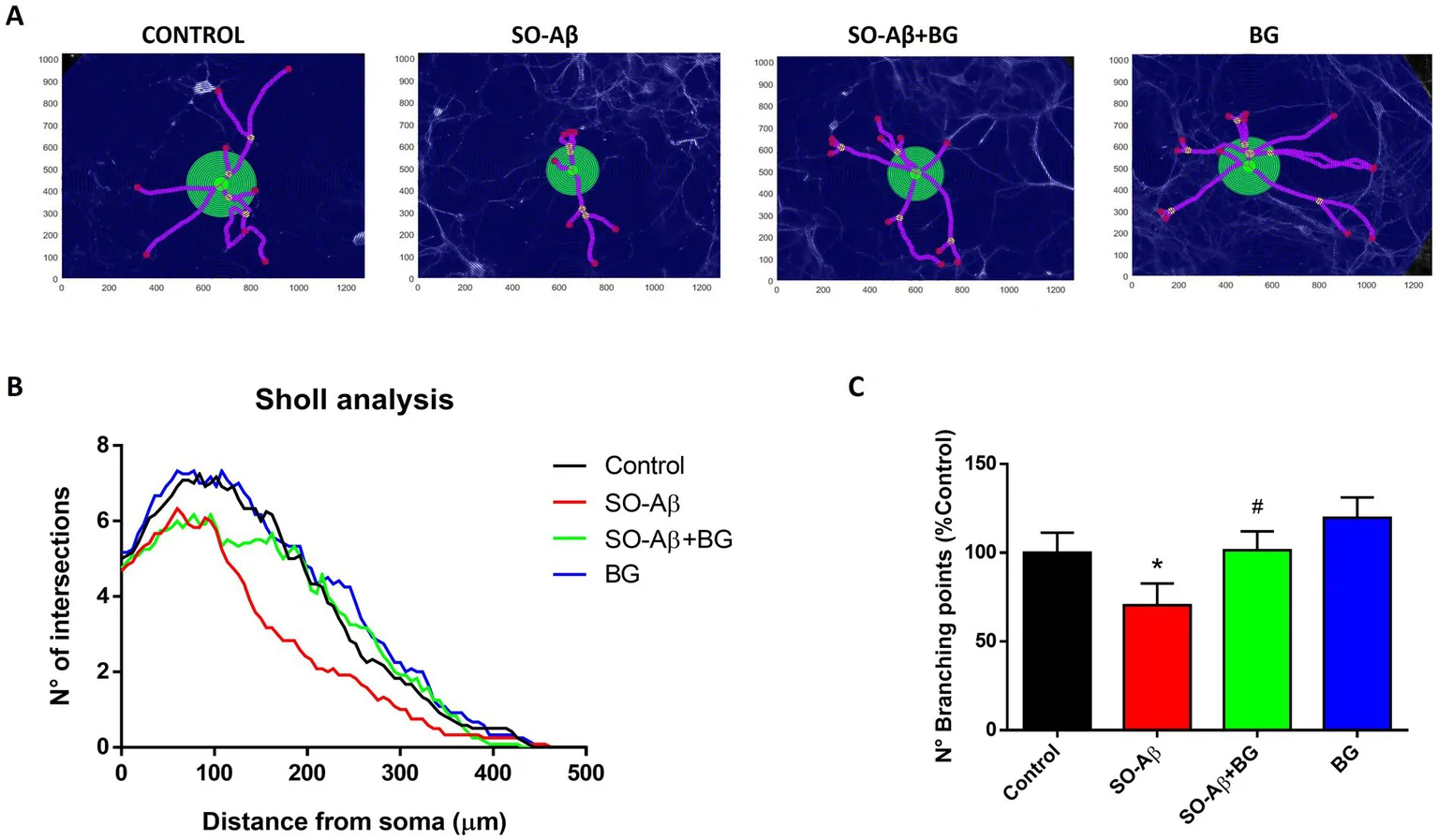
BG preserves neuronal arborization under SO-Aβ toxicity. **(A)** Representative images of hippocampal neuron morphology under chronic treatment (24 h). **(B)** Sholl analysis showing intersections of neuronal processes with concentric rings at increasing distances from the soma, in neurons treatment with SO-Aβ (0.5 μM), BG (10 μg/mL) and SO-Aβ + BG. **(C)** Quantification of branching points of hippocampal neurons (*n* = 4; *N* = 12), expressed as % of control, *p < 0.05 versus control; #*p* < 0.05 versus SO-Aβ.

The protective actions of BG on neurons suggest that its compounds induce morphological adaptations in branching complexity. Neuronal morphology and complexity are closely related to proper neuronal function and connectivity (27). To reinforce our experimental evidence regarding synaptic strength under BG treatments, we assessed neuronal arborization using Sholl analysis of MAP1B-stained neurons (Figure 4A). SO-Aβ reduced the number of intersections between neurites and Sholl rings at increasing distances from the soma (Figure 4B), indicating a loss of structural complexity. BG (10 μg/mL) preserved neuronal architecture in the presence of SO-Aβ. Quantification revealed that SO-Aβ significantly decreased total branching points (Control: 100 ± 8%; SO-Aβ: 69 ± 10%) (Figure 4C), whereas co-treatment with BG maintained branching near control values (91 ± 17%). BG alone did not significantly alter branching (112 ± 14%).

**Figure 5:**
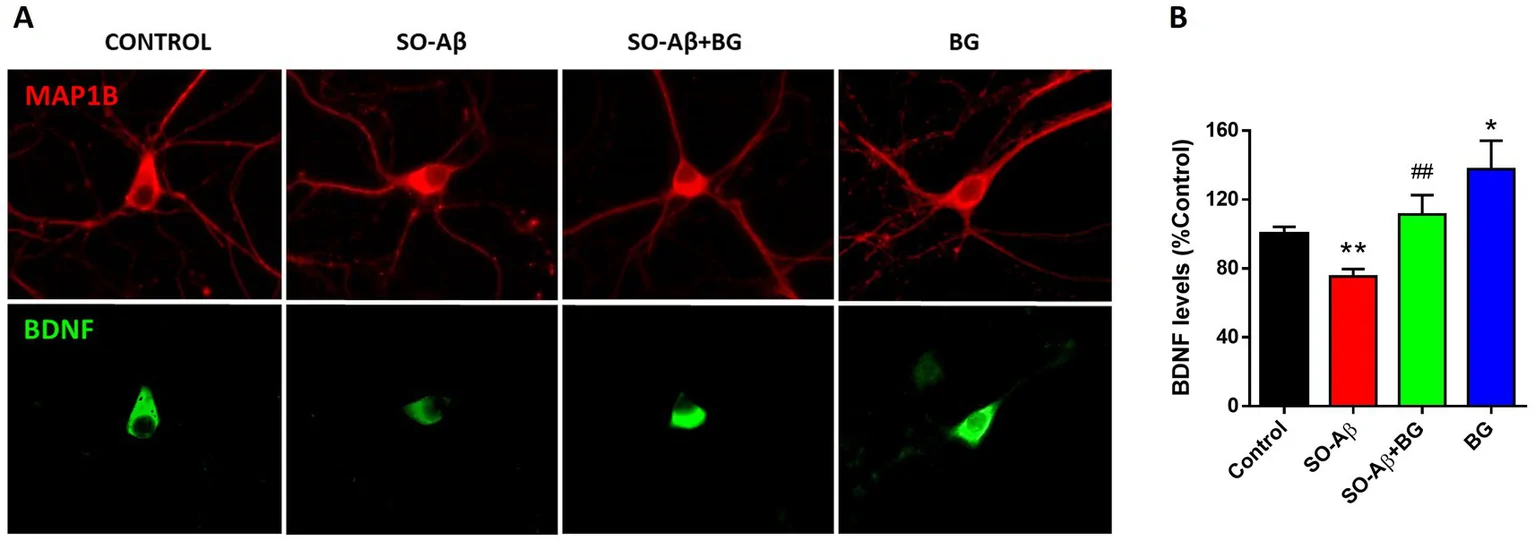
BG preserves BDNF immunoreactivity in hippocampal neurons. **(A)** Representative epifluorescence images showing MAP1B (red) and BDNF (green) after 24 h treatments with SO-Aβ (0.5 μM), BG (10 μg/mL) and SO-Aβ + BG. Scale bar: 10 μm. **(B)** Quantification of BDNF immunofluorescence, expressed as a percentage of the control (*n* = 4; *N* = 12), *p < 0.05 and **p < 0.01 versus control; ##*p* < 0.01 versus SO-Aβ.

The preservation of structural complexity suggested a possible involvement of neurotrophic factors. Brain-derived neurotrophic factor (BDNF) is a key regulator of neuronal survival and synaptic plasticity (36). We measured BDNF immunoreactivity in hippocampal neurons (Figure 5A), observing that SO-Aβ reduced BDNF levels to 25 ± 4% of control (100 ± 4%), while co-treatment with BG restored BDNF to control-like values (111 ± 11%) (Figure 5B). Remarkably, BG alone increased BDNF immunoreactivity by 37 ± 16%.

Taken together, these results indicate that 10 μg/mL BG preserves synaptic activity, structural complexity, and neurotrophic signaling in neuronal cultures. In parallel, 20 μg/mL BG was required to achieve comparable protection in hippocampal slices, consistent with the increased structural complexity and to ensure the diffusion of bioactive compounds inside of the tissue.

### 3.3 Mitochondrial dysfunction induced by SO-aβ is prevented by BG

One of the most critical components for maintaining neuronal regulation and homeostasis is the mitochondrial network, which supplies the energy required for neuronal metabolism. Our group (8, 37) and others (38, 39) have previously examined the detrimental effects of SO-Aβ on mitochondrial function. As a next step, we evaluated whether BG could preserve mitochondrial integrity by examining changes in mitochondrial membrane potential (ΔΨm) and ATP levels in mouse hippocampal slices exposed to SO-Aβ.

**Figure 6:**
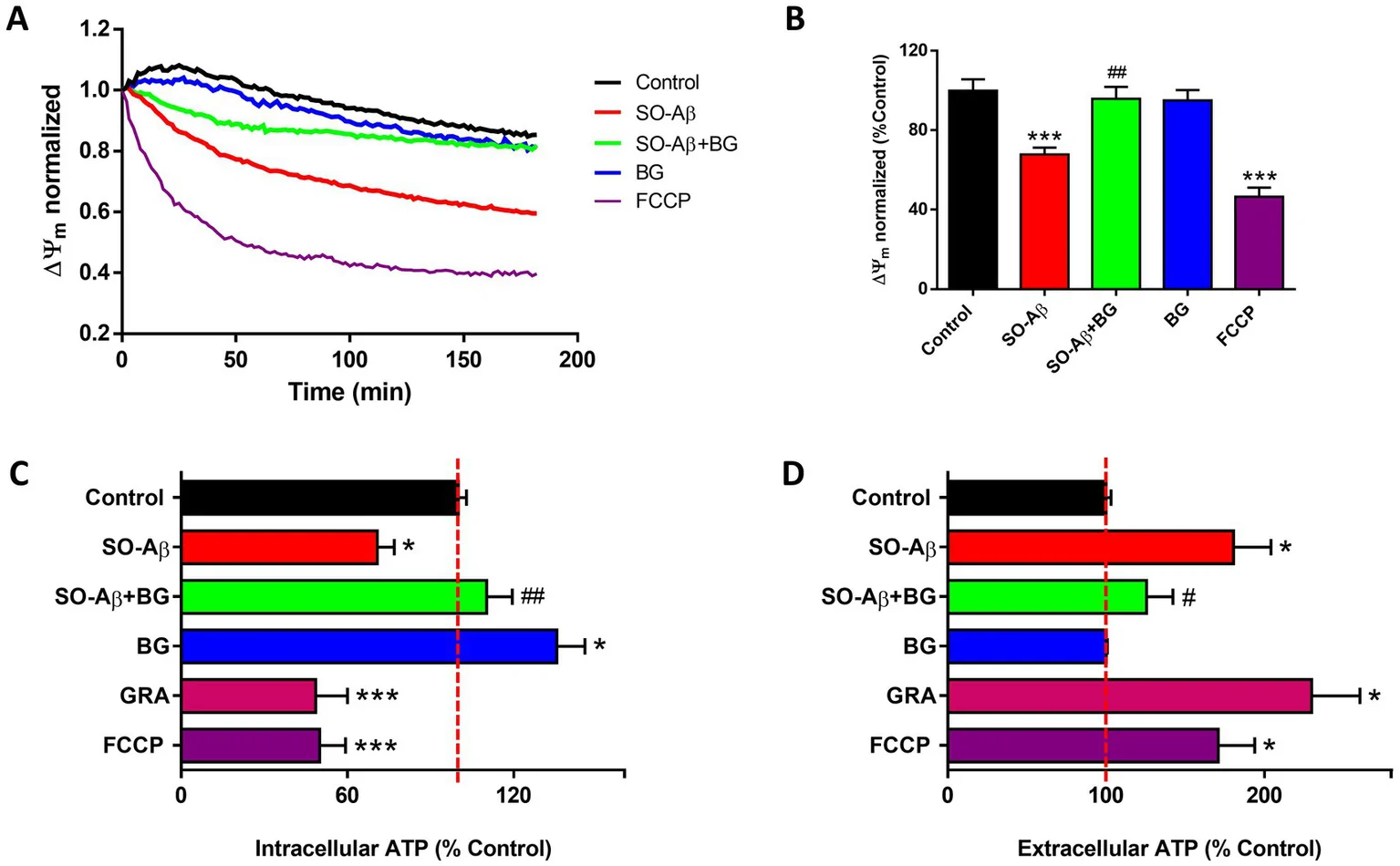
BG prevents mitochondrial alterations induced by SO-Aβ in hippocampal slices. **(A)** Time course of ΔΨm measured with JC-1 in slices treated with SO-Aβ (2.5 μM), BG (20 μg/mL), SO-Aβ + BG or FCCP (10 μM). **(B)** Quantification of ΔΨm at endpoint (*n* = 5; *N* = 16). **(C)** Intracellular ATP levels measured after 3 h of treatment with SO-Aβ (2.5 μM), BG (20 μg/mL), SO-Aβ + BG, or positive controls Gramicidin (GRA; 100 μg/mL) and FCCP (10 μM). **(D)** Extracellular ATP quantification from slice supernatants (*n* = 6). Values are expressed as a percentage of the control (*n* = 6), *p < 0.05 and ***p < 0.001 versus control; #*p* < 0.05 and ##*p* < 0.01 versus SO-Aβ.

We performed real-time ΔΨm measurements in hippocampal slices under the experimental conditions employed throughout this study. Treatments were applied at the onset of the recording period (time zero), allowing us to monitor ΔΨm changes for 180 min (Figure 6A). To quantify alterations in ΔΨm, we assessed the endpoint JC-1 fluorescence ratio (Figure 6B). SO-Aβ (2.5 μM) decreased ΔΨm by 33 ± 3% relative to control conditions, while the positive mitochondrial uncoupler FCCP (10 μM) reduced ΔΨm by 54 ± 4%. In contrast, co-incubation with BG (20 μg/mL) prevented the SO-Aβ-induced dissipation of ΔΨm, maintaining values near control (Control: 100 ± 5%; SO-Aβ + BG: 95 ± 5%). BG alone did not alter ΔΨm (95 ± 5%), indicating that BG does not compromise basal mitochondrial function.

We next quantified intracellular and extracellular ATP levels in hippocampal slices (Figure 6C). SO-Aβ treatment reduced intracellular ATP by 30 ± 6%, whereas co-treatment with BG preserved ATP levels near control (Control: 100 ± 2%; SO-Aβ + BG: 110 ± 9%). Notably, BG alone increased intracellular ATP by 35 ± 10%. As expected, the ionophores gramicidin (100 μg/mL) and FCCP (10 μM) markedly decreased intracellular ATP (52 ± 11% and 50 ± 9%, respectively). In previous work, our group proposed that SO-Aβ forms membrane pores capable of inducing ATP leakage (29). Consistent with this mechanism, we observed that SO-Aβ increased extracellular ATP by 80 ± 23% (Figure 6D). BG significantly reduced ATP leakage in SO-Aβ-treated slices (Control: 100 ± 3%; SO-Aβ + BG: 125 ± 16%). BG alone produced no detectable changes in extracellular ATP (100 ± 1%). Gramicidin and FCCP increased extracellular ATP by 129 ± 30% and 70 ± 23%, respectively.

**Figure 7:**
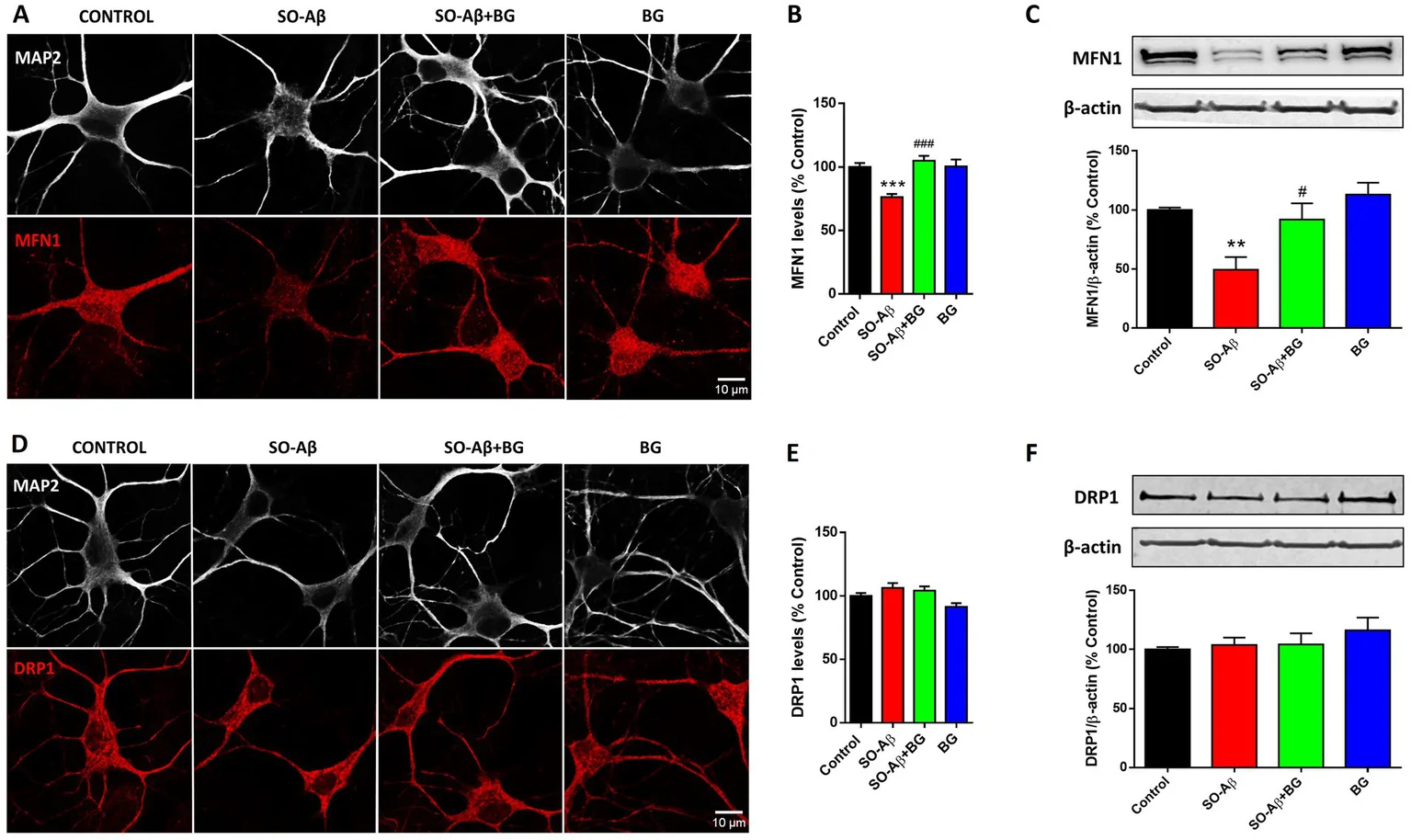
Effects of SO-Aβ and BG on the mitochondrial proteins levels MFN1 and DRP1. **(A)** Representative confocal images of hippocampal neurons treated for 24 h with SO-Aβ (0.5 μM), BG (10 μg/mL) and SO-Aβ + BG, stained for MAP2 (white) and MFN1 (red). Scale bar: 10 μm. **(B)** Quantification of MFN1 immunoreactivity (*n* = 3; *N* = 15). **(C)** Western blot of MFN1 from hippocampal slices treated for 3 h with SO-Aβ (2.5 μM), BG (20 μg/mL) and SO-Aβ + BG. The quantification of the band intensities of the upper panel is presented as a percentage of the control in the lower graph (*n* = 10). **(D)** Confocal images of neurons stained for MAP2 (white) and DRP1 (red) treated with the same conditions as A. Scale bar: 10 μm. **(E)** Quantification of DRP1 immunoreactivity (*n* = 3; *N* = 15). **(F)** Western blot quantification of DRP1 from slices, using the same conditions as in C (*n* = 10). Values are expressed as a percentage of the control, *p < 0.05, **p < 0.01 and ***p < 0.001 versus control; #*p* < 0.05 and ###*p* < 0.001 versus SO-Aβ.

To further investigate how BG affects mitochondrial health, we evaluated the expression of MFN1 and DRP1, two central regulators of mitochondrial fusion–fission dynamics (40, 41). SO-Aβ treatment (0.5 μM, 24 h) significantly decreased MFN1 immunoreactivity in hippocampal neurons (Figures 7A,B), whereas BG (10 μg/mL) preserved MFN1 levels at values comparable to control (Control: 100 ± 3%; SO-Aβ: 76 ± 2%; SO-Aβ + BG: 104 ± 3%; BG: 100 ± 5%). In hippocampal slices, SO-Aβ also reduced MFN1 levels to 51 ± 10%, while BG prevented this reduction (C: 100 ± 2%; SO-Aβ + BG: 91 ± 13%; BG: 113 ± 9%) (Figure 7C). In contrast, neither SO-Aβ nor BG produced significant alterations in DRP1 immunoreactivity in cultured neurons [Control: 100 ± 2%; SO-Aβ (0.5 μM): 106 ± 3%; SO-Aβ + BG: 104 ± 3%; BG (10 μg/mL): 91 ± 2%, 24 h] (Figures 7D,E) nor in hippocampal slices [Control: 100 ± 2%; SO-Aβ (2.5 μM): 103 ± 6%; SO-Aβ + BG: 104 ± 9%; BG (20 μg/mL): 116 ± 10%, 3 h] (Figure 7F).

Overall, these findings indicate that BG prevents SO-Aβ-induced mitochondrial dysfunction by stabilizing ΔΨm, preserving ATP production, and maintaining MFN1-dependent mitochondrial fusion, thereby supporting mitochondrial integrity during neurotoxic stress.

## 4 Discussion

Alzheimer’s disease and other dementias represent a persistent challenge for the scientific community, and the consumption of functional foods may offer effective strategies to prevent early brain alterations and promote successful aging. Here, we evaluated the neuroprotective properties of a novel nutraceutical preparation—aged garlic native to Chiloé Island (Chile), *Allium ampeloprasum* (BG)—which exhibited a robust profile of protective actions against SO-Aβ-induced neurotoxicity, preserving synaptic function and mitochondrial integrity.

We recently identified several bioactive molecules present in BG, mainly thiol derivatives such as 5-hydroxymethyl-2-furaldehyde (5-HMF), 2-propenyl sulfide (diallyl sulfide, DAS), di-2-propenyl disulfide (diallyl disulfide, DADS), 3H-1,2-dithiol-3-thione (D3T), and 4-methyl-1,2,3-trithiolane (TTL) (14). The chemical composition of BG differs substantially from that of conventional aged garlic obtained from *A. sativum* (AGE). Martínez-Casas et al. reported that the predominant compounds in AGE were DAS, DADS, and allyl methyl trisulfide (DATS), whereas D3T and TTL were not detected (42). Moreover, DAS, DADS, and DATS were found at higher concentrations in BG than in AGE. These observations indicate that uncommon organosulfur compounds such as D3T and TTL may contribute to the biological effects reported here.

Histological studies have demonstrated that AGE pretreatment prevents neuronal loss in CA1 and CA2 hippocampal regions in rats receiving Aβ injections into the ventricles, thereby attenuating memory impairments (43). These findings align with our results showing that BG preserved viability in hippocampal slices exposed to SO-Aβ. Because AGE attenuates Aβ-induced inflammatory responses, including microglial activation and IL-1β upregulation (16), our observations suggest that BG maintains mitochondrial function and limits oxidative damage under inflammatory stress. Considering that AGE-induced neuroprotection has been attributed to its sulfur-containing compounds (44, 45) the protective actions of BG are likely related to its enriched content in DAS, DADS, D3T, and TTL, molecules strongly associated with antioxidant capacity. For instance, DAS prevents gentamicin-induced lipid peroxidation in rat kidney by restoring antioxidant enzyme activity (46), while D3T acts as a potent free radical scavenger capable of activating the Nrf2 pathway (47). Using ORAC assays, Kim et al. showed that *A. ampeloprasum* exhibits greater antioxidant activity than *A. sativum*, correlating directly with its higher organosulfur content (48). Therefore, the antioxidant actions described here may reflect the proportional abundance of such compounds in BG.

Our findings demonstrate that BG confers broad protection against SO-Aβ-induced synaptic dysfunction and mitochondrial impairment, contributing to a better understanding of the mechanisms by which aged garlic preparations act as neuroprotective functional foods. At the molecular level, BG prevented SO-Aβ-induced reductions in the synaptic proteins SV2 and PSD-95 and preserved BDNF levels. These effects translated into the maintenance of neuronal morphology and spontaneous Ca^2+^ oscillations.

Importantly, beyond its protective effects under pathological conditions, BG also exerted significant beneficial actions in healthy neurons. Treatment of control preparations with BG alone significantly increased slice viability (40%), enhanced the frequency of Ca^2+^ transients (202%), elevated SV2 levels (28%), and increased intracellular ATP levels (35%). These findings indicate that BG promotes synaptic activity, neuronal metabolic support, and overall neurotrophic status even in the absence of toxic stimuli. These basal enhancements reinforce the concept that BG acts not only as a neuroprotective agent but also as a neurosupportive functional food capable of strengthening synaptic plasticity, mitochondrial function, and neuronal resilience during normal aging.

Studies focused on AGE-related neuroprotection have identified diverse mechanisms of action. Thorajak et al. demonstrated that AGE reduces cholinergic neuron loss and increases VGLUT1 levels in hippocampus, improving memory performance in rats with cognitive impairment induced by A*β* injection into the lateral ventricles (17). Similarly, oral administration of garlic essential oil enhances BDNF levels and reduces acetylcholinesterase activity in mouse hippocampus (49). These beneficial actions have highlighted neuroactive AGE compounds such as 5-HMF and DADS. Liu et al. demonstrated *in vivo* neuroprotective effects of 5-HMF against cognitive and memory impairment induced by intracerebroventricular injection of Aβ1-42. 5-HMF improved memory and learning and significantly inhibited β-secretase activity in the cerebral cortex, together with an increase in superoxide dismutase and glutathione peroxidase activity (50). DADS administration has also been shown to improve learning and memory in mice injected with Aβ_1-42_ into the lateral ventricle and to increase dendritic spine and synapse numbers in hippocampus (51). Subsequent studies showed that DADS analogs (7 k and 7I) disaggregated Aβ fibrils generated by Cu^2+^-induced aggregation and inhibited acetylcholinesterase activity with IC₅₀ values of 0.05–0.1 μM (52), and protected SH-SY5Y cells from Aβ_1-42_ toxicity by inhibiting ROS generation and restoring the Bax/Bcl-2 ratio (53). However, some evidence indicates that DADS may decrease hippocampal BDNF levels and impair memory (54), emphasizing context-dependent effects. TTL has been reported as a potent, slow-releasing hydrogen sulfide donor (55), a neuromodulator that facilitates long-term potentiation and regulates intracellular Ca^2+^ signaling (56).

Regarding neuronal morphology, thioallyl-containing garlic compounds such as DAS and DADS display neurotrophic activity (45). Although BG contains these molecules, we did not detect a significant increase in branching points in control neurons, likely due to differences in incubation time (24 h vs. 72 h) and the use of complex extracts rather than purified molecules (45). These distinctions highlight potential synergistic interactions within polyphenol- and sulfur-rich extracts. Nevertheless, the observed increases in Ca^2+^ activity, SV2 levels, ATP content, and cell viability in healthy neurons support the idea that BG exerts modulatory effects on neuronal structure and function that may contribute to enhanced physiological plasticity and synaptic efficiency.

Given the well-established link between mitochondrial dysfunction and cognitive decline (57, 58), the mitochondrial benefits of BG are particularly relevant. Previous work has shown that AGE preserves ΔΨm and ATP synthesis in ischemia models (59), improves mitochondrial function in insulin-resistant rats (60), and protects cardiac mitochondria under hyperglycemia (61). Conversely, DADS can negatively affect mitochondrial potential in cancer cells (62) and impair mitochondrial function in mouse liver (63), emphasizing context-dependent outcomes. In our study, BG preserved MFN-1 expression—a major determinant of mitochondrial fusion and network integrity—suggesting a stabilizing effect on mitochondrial dynamics and connectivity.

Despite these promising findings, several limitations must be acknowledged. The study relies on *in vitro* and ex vivo models that, while highly informative for dissecting cellular and molecular mechanisms, do not fully reproduce the systemic, vascular, inflammatory, and metabolic complexity of human Alzheimer’s disease. The concentrations of SO-Aβ and BG used in controlled preparations may not directly reflect physiological exposure or bioavailability *in vivo*. Additionally, organosulfur compounds undergo extensive metabolism, which may alter their biological activity in living organisms. Therefore, caution is required when extrapolating these results to clinical scenarios. Nevertheless, the strong effects of BG on both pathological and healthy neurons indicate relevant translational potential. The ability of BG to enhance synaptic activity, metabolic function, and neuronal resilience under basal conditions supports its consideration as a candidate functional food for promoting cognitive health and potentially delaying age-related decline. These findings justify future *in vivo* studies aimed at evaluating its bioavailability, safety, long-term effects, and cognitive benefits in animal models and, ultimately, in human populations.

In conclusion, our findings provide new evidence on the neuroprotective actions of aged elephant garlic (*Allium ampeloprasum*), native to the Chilean Patagonia, in cellular and ex vivo models of SO-Aβ toxicity. Beyond counteracting key pathological mechanisms implicated in Alzheimer’s disease, BG also enhances baseline neuronal function, increasing viability, synaptic activity, and mitochondrial performance. This dual action underscores that BG is not only a protective agent against SO-Aβ-induced damage but also a functional enhancer of neuronal physiology, highlighting its potential as a nutraceutical for promoting brain resilience and healthy aging. While further *in vivo* validation is essential, our findings suggest that the distinctive organosulfur molecules present in BG may act synergistically to produce both neuroprotective and neurosupportive effects, supporting its potential future application in preventive nutrition and brain-health strategies.

## Data Availability

The raw data supporting the conclusions of this article will be made available by the authors, without undue reservation.

## In memoriam

Patricio Varas was a clever entrepreneur, who was motivated by science and for the development of small agricultures. He was interested in providing tools for people to potentiate their small business in places far away in other parts of the country like Chiloe Island. We are so grateful for his support to the science. We dedicate this work to the memory of Patricio Varas (RIP), from MeliMei company, for his contribution to the research and development of aged garlic in Chile.
