# Correction: Unraveling the neuroprotective mechanisms of elephant black garlic extract against beta amyloid peptide-induced neurotoxicity

**DOI:** 10.3389/fnut.2026.1960775

**Published:** 2026-08-20

**Authors:** Javiera Gavilan, Jessica Panes-Fernández, Aníbal Araya, Claudia Pérez-Manríquez, José Becerra, Patricio Varas, Gustavo Moraga-Cid, Gonzalo E. Yévenes, Jorge Fuentealba

**Affiliations:** 1 Departamento de Fisiología, Facultad de Ciencias Biológicas, Universidad de Concepción, Concepción, Chile; 2 Laboratorio de Química de Productos Naturales, Facultad de Ciencias Naturales y Oceanográficas, Universidad de Concepción, Concepción, Chile; 3 Agrícola Melimei, Ancud, Chile; 4 Millennium Nucleus for the Study of Pain (MiNuSPain), Concepción, Chile; 5 Centro de Investigaciones Avanzadas en Biomedicina (CIAB-UdeC), Universidad de Concepción, Concepción, Chile

**Keywords:** aged garlic, *Allium ampeloprasum*, Alzheimer's disease, mitochondrial dysfunction, neuroprotection, soluble oligomers of beta-amyloid peptide (SO-aβ), sulphurated compounds, synaptic failure

In the **Acknowledgments**, the following sentence was omitted, “JG acknowledges financial support from the Universidad de Concepción and the Vicerrectoría de Investigación y Desarrollo (VRID) through the Postdoctoral Grant No. 202500025P”. The correct statement should read as, “The authors thank Ixia Cid, Jocelin Gonzalez, and CMA BIO-BIO for their technical assistance. JG acknowledges financial support from the Universidad de Concepción and the Vicerrectoría de Investigación y Desarrollo (VRID) through the Postdoctoral Grant No. 202500025P.”

The original version of this article has been updated.

