# Supplementary figures and images for: Unraveling the neuroprotective mechanisms of elephant black garlic extract against beta amyloid peptide-induced neurotoxicity
Source: Front Nutr. 2026 Jan 16;12:1725284. doi: 10.3389/fnut.2025.1725284 (PMC12857319; doi:10.3389/fnut.2025.1725284)

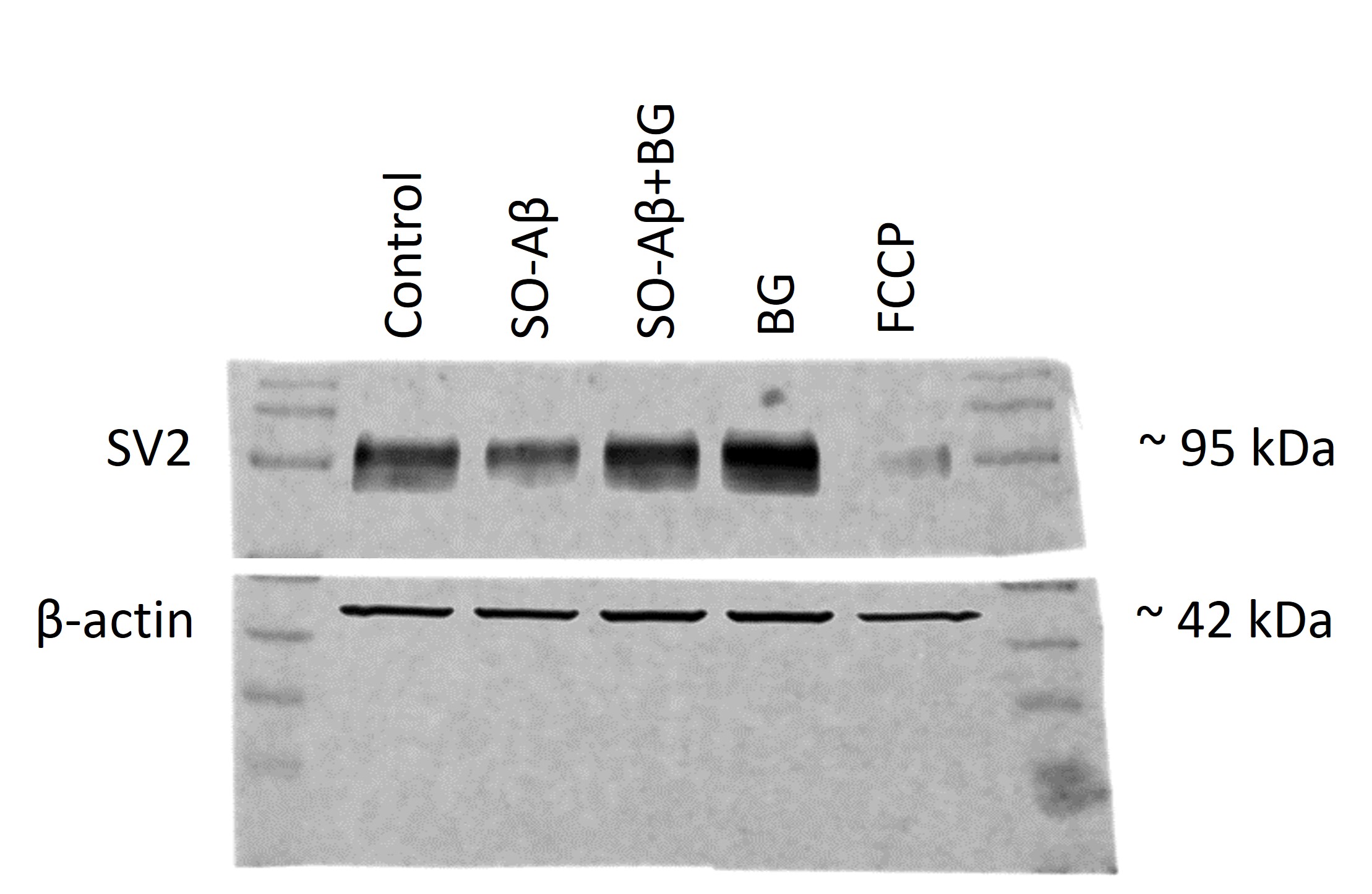

Supplement: Supplementary file 1 [file Image_1.jpeg]

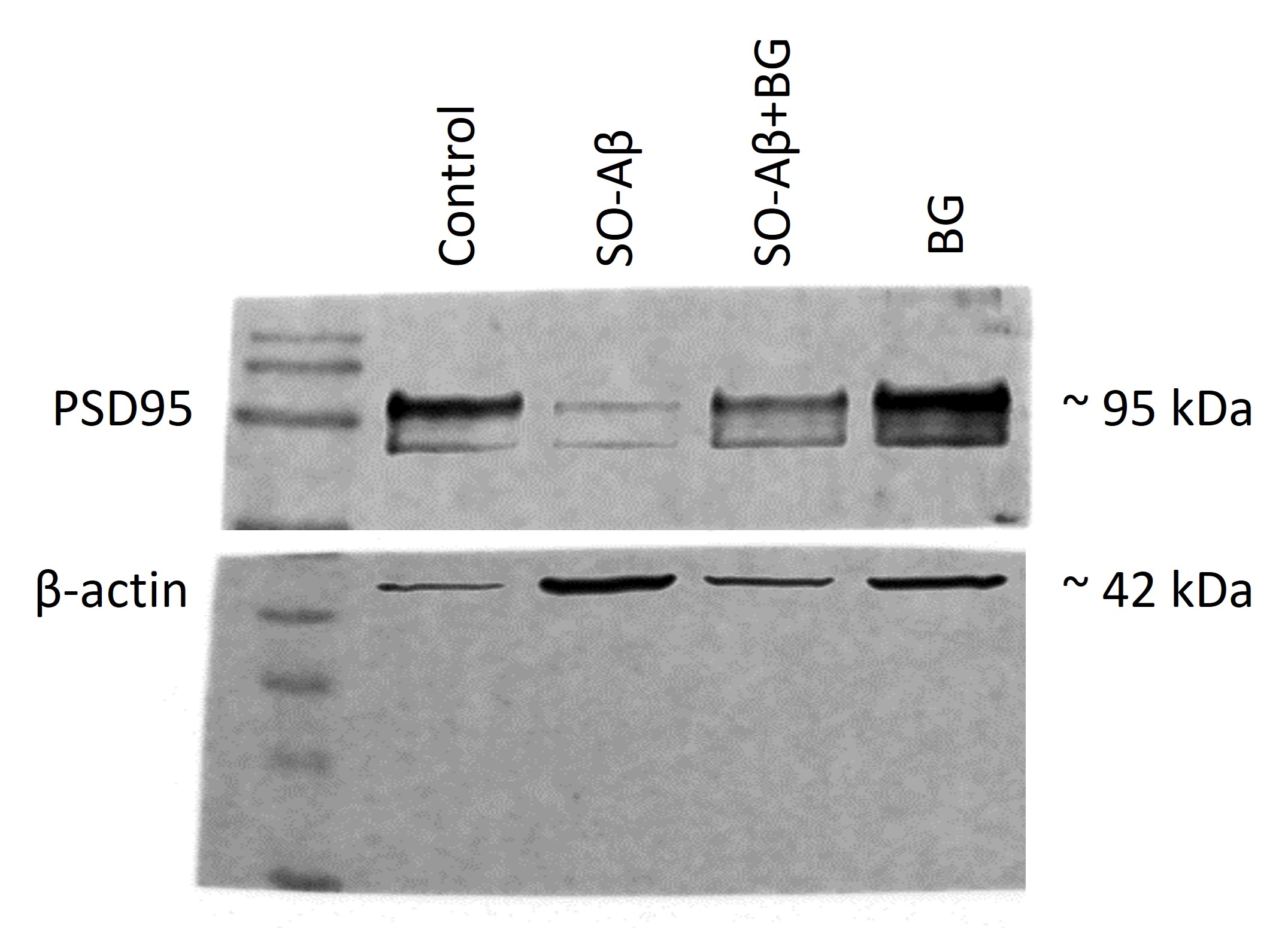

Supplement: Supplementary file 2 [file Image_2.jpeg]

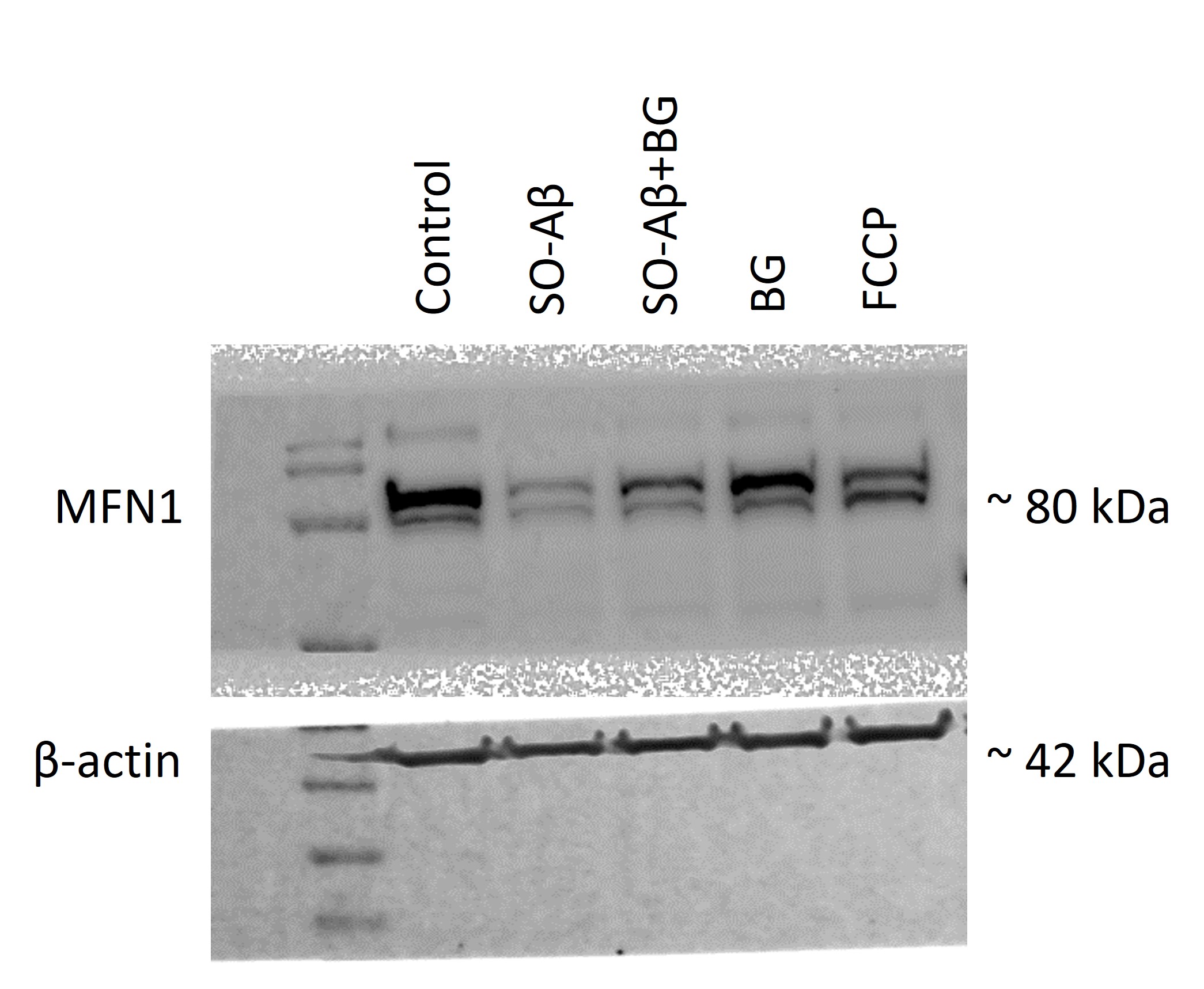

Supplement: Supplementary file 3 [file Image_3.jpeg]

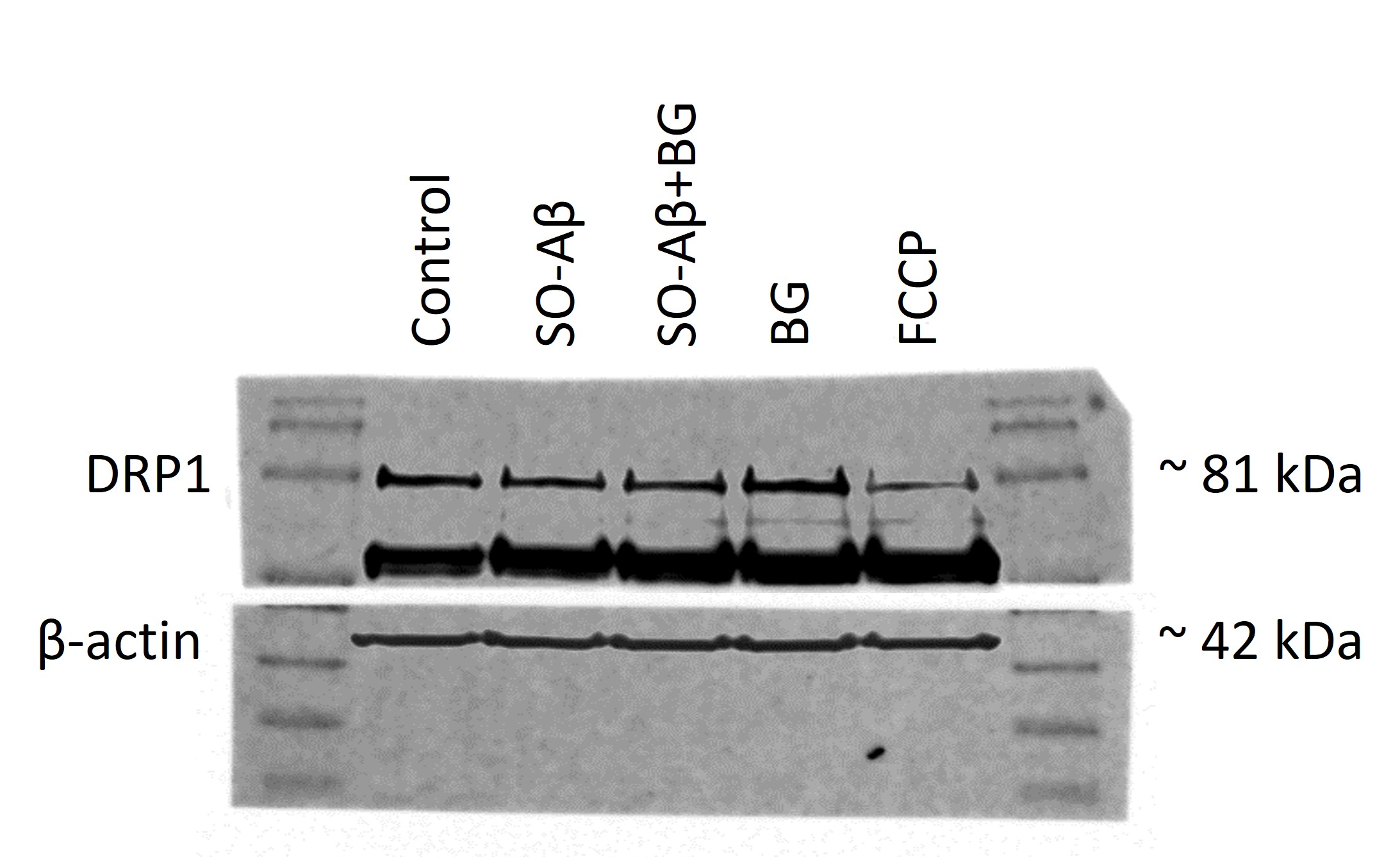

Supplement: Supplementary file 4 [file Image_4.jpeg]
